# Tissue-specific roles of regulatory T cells: mechanisms of suppression and beyond along with emerging therapeutic insights in autoimmune indications

**DOI:** 10.3389/fimmu.2025.1650451

**Published:** 2025-08-26

**Authors:** Bat-Erdene Jugder, Eunchong Park, Lijuan Du, Chetan Jawale, Nikolay Popov, Zengli Guo, Kyle J. Bednar, Tatiana Ort

**Affiliations:** ^1^ Bioscience Immunology, Research and Early Development, Respiratory and Immunology, Biopharmaceuticals R&D, AstraZeneca, Waltham, MA, United States; ^2^ Bioscience Immunology, Research and Early Development, Respiratory and Immunology, Biopharmaceuticals R&D, AstraZeneca, Gaithersburg, MD, United States; ^3^ Biologics Engineering, Oncology R&D, AstraZeneca, Gaithersburg, MD, United States; ^4^ Bioscience Immunology, Research and Early Development, Respiratory and Immunology, Biopharmaceuticals R&D, AstraZeneca, Cambridge, United Kingdom

**Keywords:** Tregs (regulatory T cells), tissue Tregs, autoimmunity, immune tolerance, CAR-Treg, Treg plasticity, tissue repair

## Abstract

Regulatory T cells (Tregs) are central to immune homeostasis and controlling inflammation through multiple mechanisms, however, recent discoveries and advances in technology reveal that Tregs exert a diverse array of functions beyond mere immunosuppression, adapting uniquely to the specialized environments of tissues. This review delves into the multifaceted, tissue-specific mechanisms of Tregs, highlighting their roles in tissue repair, inflammatory modulation, and tolerance maintenance. We explore the developmental, functional, and metabolic pathways that drive Treg specialization across distinct organs, such as the central nervous system, gastrointestinal tract, joints, skin, and lungs, and examine how these insights advance the design of novel, targeted therapies for autoimmune and inflammatory disorders. This review will emphasize non-suppressive functions, discussing how Tregs can be harnessed in therapeutic applications tailored to specific tissue microenvironments, offering a promising new direction for the treatment of autoimmune diseases.

## Introduction

Regulatory T cells (Tregs) are pivotal in maintaining immune homeostasis, primarily recognized for suppressing effector immune cell responses to enforce self-tolerance. Defined by Foxp3 expression, Tregs have traditionally been viewed as key regulators of immune overactivity and their dysfunction is linked with many autoinflammatory diseases. Foxp3 is a lineage-defining transcription factor that is essential for Treg development, stability, and suppressive function. Autoimmune diseases affect millions of patients globally with currently available treatments offering limited disease modification for a subset of patients known as the “efficacy ceiling”. This therapeutic ceiling reflects a failure to account for the specialized adaptations of tissue-resident Tregs, which operate in distinct microenvironments and perform functions beyond classical suppression. However as these diseases are being studied more in depth for mechanisms of actions, a broader perspective on Treg functionality is emerging. Recent studies reveal that Tregs exhibit tissue-specific roles far beyond immunosuppression, adapting to the distinct microenvironments of organs such as the gastrointestinal (GI) tract, lungs, skin, joints, central nervous system (CNS), and adipose tissues. This paradigm shift highlights the importance of non-suppressive functions, such as tissue repair, metabolic regulation, and stromal crosstalk, that are increasingly recognized as central to Treg-mediated tissue homeostasis. These functions are particularly relevant in autoimmune diseases, where immune dysregulation often coincides with tissue damage and impaired regeneration.

Treg heterogeneity stems from their developmental origins and local adaptation. Thymus-derived Tregs establish baseline tolerance, while peripherally induced Tregs respond to tissue-specific signals, such as microbial metabolites or injury cues. As an example of this beyond IL-10-mediated suppression, Tregs facilitate lung epithelial repair via amphiregulin (AREG) and enhance adipose insulin sensitivity through IL-33 signaling. However, chronic inflammation or aging can destabilize Treg function, potentially exacerbating disease. Understanding these tissue-tailored mechanisms is essential for therapeutic advancement.

This review synthesizes current insights into Treg specialization across tissues, highlighting their non-suppressive functions and the development of targeted therapies. From engineered Tregs for joint-specific inflammation to strategies enhancing gut Treg stability, we explore how these approaches promise precise interventions for autoimmune disorders, redefining Tregs as versatile mediators of immunity and tissue homeostasis. While murine studies have provided deep mechanistic insights into Treg specialization, translating these findings into human therapies remains challenging due to limited understanding of tissue-resident Treg phenotypes, homing signatures, and stability requirements in humans. Addressing these gaps is critical for designing next-generation, tissue-targeted Treg therapies with durable efficacy in autoimmune indications.

## Tissue Tregs

### Differentiation and functional plasticity across tissues

Tregs exhibit remarkable adaptability to diverse tissue microenvironments, integrating developmental, transcriptional, epigenetic, and metabolic cues to preserve immune tolerance and support tissue homeostasis. This plasticity enables them not only to suppress immune responses but also to participate in tissue repair, regeneration, and metabolic regulation.

Tregs can originate from the thymus (tTregs, also referred to as natural Tregs or nTregs) or differentiate peripherally from naïve CD4^+^ T cells (pTregs). Helios^+^ tTregs in the gut develop independently of microbiota-derived signals, while RORγt^+^ pTregs require MHCII-dependent antigenic stimulation and costimulatory pathways (CD28, ICOS) for their differentiation and maintenance (1). This distinction suggests that tissue-specific signals influence lineage commitment via distinct molecular mechanisms. Studies have further demonstrated that nTregs, generated in the thymus via high-avidity TCR selection, primarily enforce self-tolerance, whereas pTregs, induced at mucosal interfaces, are crucial for tolerance to commensals and dietary antigens (2). Additionally, intestinal pTregs require microbial-derived metabolites such as short-chain fatty acids (SCFAs) and retinoic acid to sustain their suppressive function (3, 4). This suggests that environmental factors critically shape peripheral Treg induction and stability.

**Figure 1:**
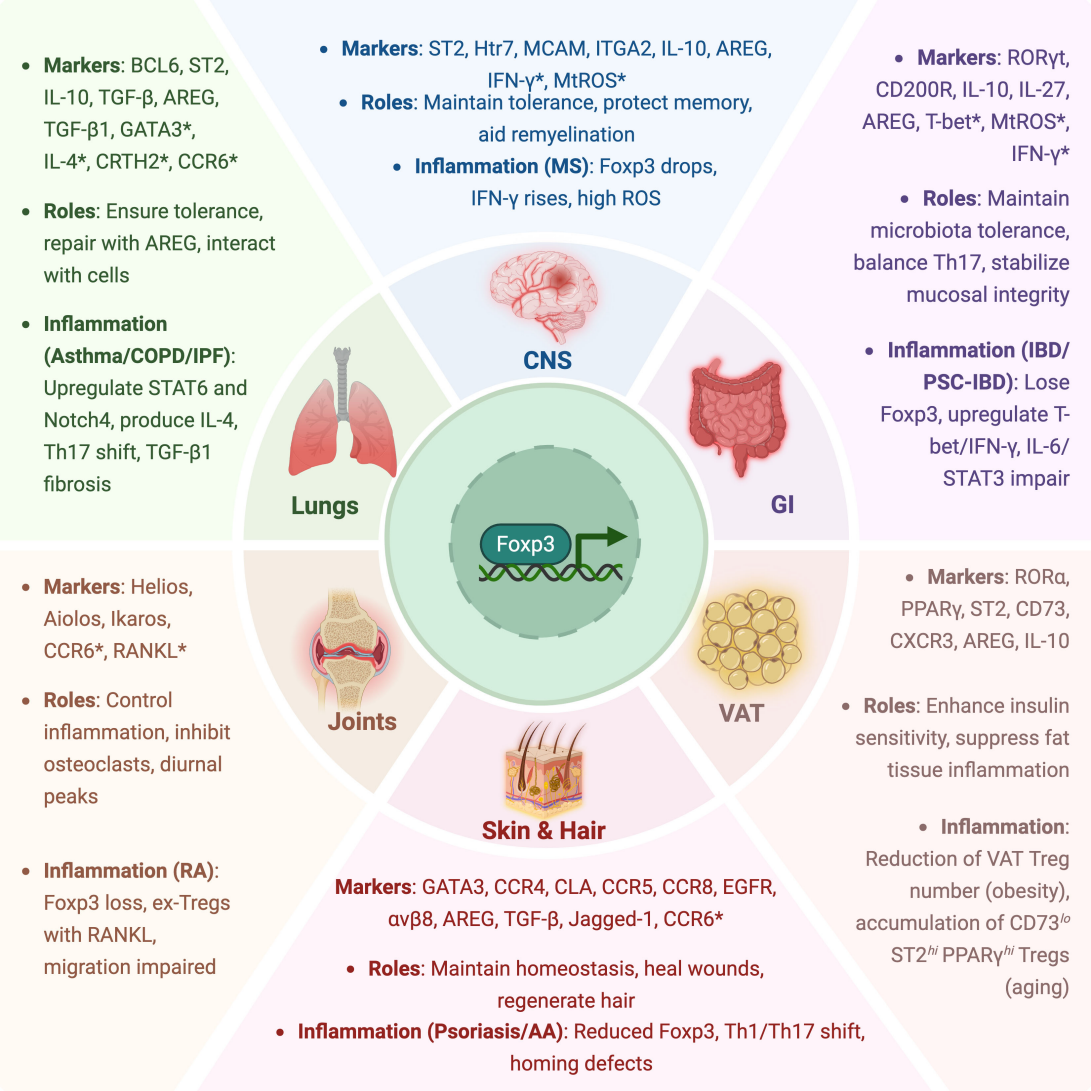
Tissue-specific adaptations and inflammatory dysregulation of Tregs. This schematic illustrates the specialized roles of Tregs across major tissue compartments, including the gastrointestinal (GI) tract, lungs, central nervous system (CNS), joints, skin and hair, and visceral adipose tissue (VAT). For each tissue, representative markers, key functional roles, and relevant inflammatory contexts are highlighted. Asterisks (*) indicate markers associated with Treg dysfunction, instability, or pro-inflammatory conversion under disease conditions.

Treg plasticity allows adaptation to local immune environments, but excessive inflammation, tissue damage, or dysregulation of microbiota for instance can compromise immune tolerance. Epigenetic programs reinforce Treg lineage stability while allowing functional shifts, such as the acquisition of Th1-, Th2-, or Th17-like phenotypes under inflammatory conditions (5)​. However, loss of Foxp3 expression and conversion into ex-Tregs can drive pathogenic immune responses in chronic inflammation as seen in autoimmunity. Aging imposes additional constraints on Treg plasticity. Single-cell transcriptomics was used to demonstrate that aging leads to metabolic and transcriptional shifts, including a decline in CD150^hi^ precursor Tregs and a metabolic switch from oxidative phosphorylation to glycolysis (6). These findings highlight how environmental factors such as inflammation and metabolic stress dynamically reshape the Treg compartment across tissues and disease microenvironments. Treg specialization also extends beyond immune suppression, as tissue-adapted Tregs can promote wound healing and metabolic regulation. For example, IL-33–responsive Tregs in visceral adipose tissue (VAT) support insulin sensitivity (7), while AREG-expressing Tregs contribute to epithelial repair in the lungs, gut and skin (8)​. These adaptations and their functional implications across different tissue compartments are summarized in **Figure 1**.

Distinct transcriptional networks enforce Treg specialization across tissues. A comprehensive regulatory network was mapped, showing that Foxp3 alone is insufficient for Treg adaptation (9). Instead, it cooperates with tissue-specific transcription factors such as T-bet (Th1-like Tregs), PPARγ (adipose Tregs), GATA3 (skin Tregs), and RORγt (intestinal Tregs) to drive tissue-specific programs​. At the epigenetic level, TNFR2 costimulation was shown to promote Treg differentiation into non-lymphoid, tissue-resident effector cells, independent of classical Th-like polarization (10). Their findings suggest that TNFR2 signaling could be leveraged to generate tissue-adapted Tregs for therapeutic applications​. Additional studies have noted that Tregs co-opt lineage-defining transcription factors to regulate immune responses in specific tissues. T-bet^+^ Tregs suppress Th1-driven inflammation​, IRF4^+^ Tregs counteract Th2-mediated immunity​, and STAT3-dependent RORγt^+^ Tregs enforce gut homeostasis (5, 11, 12)​. This demonstrates that molecular cooperation is a key strategy enabling Tregs to balance immune tolerance with environmental adaptation. Further epigenetic controls have revealed the role of BATF, IRF4, and Blimp-1 in driving effector-like Treg programs in tissues such as the skin and gut (13).

### Treg stability, plasticity, and non-suppressive functions in tissue environments

Treg specialization is reinforced by chromatin remodeling and histone modification (10). Additional regulators like BATF, Blimp-1, and IRF4 play vital roles in establishing tissue-specific effector-like Treg states (13). These modifications are heritably encoded by epigenetic marks, particularly within the Foxp3 locus, which includes conserved non-coding sequences (CNS0–3) and the Treg-specific demethylated regions (TSDR). CNS2 is crucial for stable *FOXP3* transcription as it recruits key transcription factors such as CREB, Ets-1, Stat5, Runx, c-Rel, and Foxp3 itself (14). Notably, autoimmune-associated SNPs are disproportionately localized within CpG hypomethylated regions that define naïve Treg-specific regulatory elements, suggesting that disruption of Treg epigenetic identity may underlie genetic susceptibility to immune dysregulation (15). CNS0 and CNS3 function as early epigenetic gates, recruiting factors like BRD9, MLL4, and STAT5 (16, 17) during lineage induction, while CNS1 is particularly important for TGFβ-induced pTreg differentiation via AP-1, Foxo1, Smad3, and Hhex (18).

The FOXP3 protein is also tightly regulated post-translationally through acetylation and ubiquitination, which affects its stability and suppressive function (19). Histone deacetylases (HDACs) such as HDAC6, HDAC9, and HDAC10, as well as sirtuin 1, influence Treg function, and their inhibition can enhance Treg suppressive capacity (20–23). HDAC7, which interacts with FOXP3 and TIP60, is especially critical in maintaining pTreg function and limiting neuroinflammation (24). PRC2, polycomb repressive complex 2, particularly its catalytic subunit EZH2, is necessary for establishing an epigenetic landscape that supports effector Treg survival; EZH2 deficiency disrupts this balance and contributes to autoimmune pathology in both rheumatoid arthritis (RA) and inflammatory bowel disease (IBD) models (25, 26).

Cytokines within the tissue microenvironment exert powerful control over Treg stability and functional orientation. IL-2 is indispensable for Treg survival, FOXP3 maintenance, and STAT5-driven transcriptional programming (27). In combination with TGF-β, it promotes *FOXP3* induction and pTreg differentiation, with Smad3 and STAT5 binding the FOXP3 CNS regions to stabilize lineage fate (28, 29). Conversely, inflammatory cytokines such as IL-6 and IL-1β destabilize Tregs. IL-6 promotes STAT3 activation, which enhances Th17 differentiation while antagonizing Treg polarization (30, 31). It can also disrupt FOXP3-EZH2 interactions (32), and its inhibition in RA patients has been shown to restore Treg homeostasis (33). IL-1β interferes with Treg development by engaging HIF-1α/mTOR pathways and promotes Th17-like conversion (34), while IL-33 enhances Treg stability and limits inflammatory cytokine acquisition (35).

Among TNF family cytokines, TNFR2 signaling emerges as a particularly important axis in Treg stability. Tregs preferentially express TNFR2, and its ligation helps maintain the demethylated state of the *FOXP3* promoter, thereby preserving FOXP3 expression and protecting Tregs from inflammatory conversion (36). Genetic studies have linked *TNFRSF1B* (gene encoding TNFR2) polymorphisms to susceptibility in RA and IBD (37), and murine models show that TNFR2 deficiency exacerbates autoimmune responses across multiple disease settings (38–41). These findings indicate TNFR2 as potential pathway to drive a selective Treg-targeted immunotherapy (42).

Despite their lineage-defining stability, Tregs retain transcriptional flexibility and can co-express lineage-specifying transcription factors such as T-bet, GATA3, Bcl6, and RORγt; normally associated with Th1, Th2, Tfh, and Th17 cells, respectively (43–46). In autoimmune microenvironments, this plasticity can evolve into functional fragility, a state in which Tregs continue to express FOXP3 but lose suppressive capacity while acquiring effector-like traits, including secretion of IFN-γ or IL-17 (47). IL-12-driven IFN-γ expression is one defining marker of such dysfunctional Tregs (48). Aging exacerbates this process by shifting Treg metabolism from oxidative phosphorylation to glycolysis and depleting precursor populations such as CD150^hi^ Tregs, further compromising Treg durability and repair potential (6).

Importantly, tissue-resident Tregs fulfill diverse non-suppressive functions that extend beyond immune regulation. These include promoting epithelial repair through AREG secretion, orchestrating angiogenesis (49), and modulating tissue-specific metabolic inflammation (50). Tregs also engage in direct interactions with stromal elements such as epithelial cells, fibroblasts, and endothelial cells to reinforce barrier function and maintain tissue architecture. Recent studies highlight that these reparative and regulatory circuits can be therapeutically leveraged, and delineated the uncoupling of suppressive and reparative functions (51), while the recent study (52) reviewed biomaterial-based strategies to selectively augment Treg-mediated repair without broad immunosuppression.

In summary, tissue-resident Tregs are deeply shaped by their local environment, acquiring distinct transcriptional and functional profiles that enable them to balance immune tolerance with organ-specific repair and regeneration. Yet this specialization renders them acutely vulnerable to destabilization under chronic inflammatory, metabolic, or aging-associated stress. Therapeutic strategies that restore or reinforce Treg stability and plasticity, while preserving their non-suppressive functions, offer a promising avenue for treating autoimmune and inflammatory diseases.

## Tissue-specific roles of Tregs in autoimmune indications

### Tregs in the GI tract

#### Tissue-specific adaptation and immune homeostasis

The intestinal environment presents a unique immunological challenge, constantly exposed to dietary antigens, commensals, and occasional pathogens. To maintain tolerance and barrier integrity, the gut harbors a diverse population of Tregs, primarily composed of Helios^+^ tTregs and RORγt^+^ pTregs, with the latter being microbiota-dependent (1). ICOS and CD28 costimulatory signaling is essential for the maintenance of RORγt^+^ Tregs, as their depletion results in a loss of microbiota tolerance and increased colitis severity​. The microbiota plays a critical role in shaping gut Treg populations, as germ-free mice exhibit significantly reduced pTregs, highlighting their dependence on microbial cues. Bile acid metabolites have emerged as key regulators of gut Treg homeostasis. Two derivatives of lithocholic acid, 3-oxoLCA and isoalloLCA, regulate T cell differentiation in the intestine (53). While 3-oxoLCA directly inhibits Th17 differentiation via RORγt binding, isoalloLCA enhances Treg differentiation by promoting mitochondrial reactive oxygen species (mtROS) production and stabilizing *Foxp3* expression​. Specific bacterial strains also influence gut Treg differentiation through their metabolites and SCFAs. Moreover, specific bacterial strains such as *Clostridia* and *Bacteroides fragilis* promote pTreg development, reinforcing microbiota-Treg crosstalk (54)​. Furthermore, *Bacteroides* species can generate SCFAs, such as butyrate, which enhance pTreg differentiation by modulating histone acetylation at the *Foxp3* locus, the importance of which is described above​. These findings highlight how gut microbiota fine-tune intestinal immune tolerance by balancing Th17 and Treg populations.

#### Dysregulation in IBD and primary sclerosing cholangitis-IBD

In IBD, dysregulated Tregs contribute to uncontrolled intestinal inflammation. The colonic lamina propria harbors a significant population of RORγt^+^ Tregs, which are crucial for maintaining gut homeostasis by suppressing excessive Th17-driven inflammation (55). However, in IBD patients, FOXP3^+^ Tregs show altered transcriptional profiles with reduced *IL10* expression, impairing their regulatory capacity (56). Unlike IL-10, which broadly suppresses inflammation, IL-27, produced by gut-resident Tregs, plays a critical role in selectively suppressing Th17-driven pathology while preserving mucosal defense (55). Notably, microbiota-driven induction of IL-27^+^ Tregs is essential for regulating gut inflammation without compromising systemic immunity​. Mice lacking Treg-derived IL-27 exhibit exacerbated gut inflammation with heightened Th17 responses, highlighting its regulatory role in immune homeostasis​ in the GI tract. However, IL-27 plays a context-dependent role in inflammation: in the gut, Treg-derived IL-27 can limit Th17 responses and ameliorate colitis, whereas in other tissues such as the CNS or joints, IL-27 may exert pro-inflammatory effects by promoting pathogenic Th1 or Tr1 responses (57). Impacts of immune microniches on Treg function in the lamina propria have extensively been studied. The colonic lamina propria acts as a crucial site for Treg-mediated immune regulation. Spatial transcriptomic analyses have identified key receptor-ligand interactions, such as CD200-CD200R, that govern Treg stability in the gut (56). The presence of CD103^+^ SIRPα^+^ dendritic cells and CD206^+^ macrophages supports the maintenance of effector Tregs, reinforcing localized tolerance​. However, in IBD, inflammation disrupts these microniches, leading to the loss of compartmentalized Treg function and dominance of inflammatory effector T cells (56). Moreover, ILC3s maintain gut tolerance by promoting microbiota-specific RORγt^+^ Tregs via MHCII antigen presentation and IL-2 competition, preventing Th17 conversion. In IBD, disrupted ILC3-Treg interactions were found to reduce RORγt^+^ Tregs, driving inflammation (58).

Metabolic regulation has emerged as a critical determinant of Treg stability in the gut. Among several inflammatory metabolites, succinate plays a particularly disruptive role. In the context of IBD, elevated succinate levels impair Treg function by interfering with FOXP3 succinylation, a protective post-translational modification that normally shields FOXP3 from proteasomal degradation. Succinate suppress succinyl-CoA synthesis, which removes this stabilizing mark, leading to FOXP3 ubiquitination and degradation. This metabolic interference contributes to the loss of lineage stability and suppressive capacity of Tregs in inflamed intestinal tissues (59).

Another IBD-related condition where Treg dysfunction is a hallmark is primary sclerosing cholangitis-associated IBD (PSC-IBD), a severe colonic inflammation linked to biliary disease. In PSC-IBD, gut-homing Tregs were shown to display impaired stability, with increased expression of inflammatory markers such as T-bet and IFN-γ, contributing to disease progression (60)​. The liver-gut axis plays a critical role in regulating Treg function in PSC-IBD as demonstrated by bile acid metabolites modulating their suppressive capacity via the FXR signaling (53)​. A recent single-cell study highlights dysfunctional Treg differentiation and an imbalance in the Treg/Th17 axis as key contributors to PSC pathogenesis (61). It suggests that biliary epithelial cells and hepatic monocytes drive a Th17-polarizing microenvironment, exacerbating inflammation. Furthermore, clinical studies have demonstrated that PSC is characterized by reduced Treg frequencies, low CD39 expression, and elevated IL-6 levels. IL-6 exposure enhances STAT3 activation in Tregs, impairing their inhibitory function and exacerbating inflammation (62). In line with these findings, a recent study proposes a clinical trial using autologous polyclonal Tregs for PSC-IBD (63). Given these insights, restoring Treg homeostasis through microbial-derived metabolites, cytokine modulation, or bile acid-targeted therapies represents a promising approach for treating both IBD and PSC-IBD.

#### Therapeutic strategies and translational advances

Therapies aimed at restoring gut Treg function are advancing rapidly. Several platforms are being explored to engineer polyclonal or antigen-specific Tregs to re-establish tolerance in IBD and related disorder. A few advances include engineering antigen-specific Tregs to restore immune tolerance and repair gut inflammation in IBD (64), while others are utilizing the advances in gene-modification to create chimeric antigen receptor Tregs (CAR-Tregs) in a more tailored approach to their binding to specific antigens of interest for particular autoimmune diseases, including ulcerative colitis (UC) and Crohn’s disease (CD). Others are utilizing the plasticity of CD4^+^ T cells to pioneering approaches to manipulate suppressive Tregs, with potential applications in GI-related autoimmunity. Further, there was a recent publication on IL23R-specific CAR-Tregs for CD, leveraging IL-23’s role in disease pathology to enhance targeted immune suppression​ (65). Similar to leveraging advances in gene-modification and lessons learned from oncology, TCR-engineered Tregs have been explored in targeting type 1 diabetes, with broader implications for autoimmune conditions affecting mucosal tissues​. Meanwhile, there are advances in engineered CAR-Tregs equipped with molecular features that enhance their stability, persistence, and suppressive function, to maintain Treg stability, persistence, and function under inflammatory conditions through a novel multi-modality platform designed to enhance Tregs in autoimmune conditions (64, 66). These modification and feats in engineering represent the forefront of Treg-cell based innovation in gastroenterology, aiming to harness the immunoregulatory potential of Tregs for durable disease remission and immune homeostasis in IBD.

Notably, a recent first-in-human feasibility study (TRIBUTE) is evaluating the safety and tolerability of a single-dose infusion of ex vivo-expanded autologous tTreg (TR004) in patients with moderate-to-severe CD who have failed at least two prior therapies. This trial tracks infused Tregs using deuterium labeling to assess their persistence, gut homing, and stability with the goal of restoring immune tolerance and overcoming mucosal Treg dysfunction (67). In parallel, small molecule and cytokine-based approaches, including IL-2 muteins, FXR agonists, and bile acid mimetics, are being explored to support endogenous Treg stability and gut localization (68–71).

### Tregs in lungs

#### Lung-resident Tregs in immune regulation and tissue repair

In steady-state lungs, Tregs function at the interface of immune tolerance and tissue maintenance, adapting to the constant antigenic and mechanical stressors of the respiratory system. They play dual roles: restraining immune responses to inhaled antigens and orchestrating tissue repair following injury. Lung-resident Tregs are enriched in the parenchyma and airway-associated lymphoid tissue, where they suppress harmful immune responses via IL-10 and TGF-β and interact with a diverse set of cell types including epithelial, stromal, and mesenchymal populations (72–74).

Importantly, lung Tregs are equipped with unique non-suppressive programs. The tissue repair function of Tregs was first reported by Dr. King group using acute lung injury (ALI) model (75). They found that the transfer of CD4^+^ CD25^+^ splenic T cells, but not other T cell subsets, could promote the resolution of tissue damage in immune deficient mouse models. Tissue damage could induce the production of IL-18 and IL-33, which have been shown to promote AREG production by Tregs (8). AREG is a growth factor that stimulates the proliferation and differentiation of epithelial cells and helps to repair damaged lung tissue (76, 77). AREG-producing lung Tregs are found to accumulate in the infection site of influenza and regulate the fibroblast growth factor production in Col14^+^ mesenchymal cells for the regeneration of alveolar structure (78). Additionally, lung Tregs can recognize tissue-specific antigens and respond to self-reactivity during sterile inflammation, further highlighting their unique adaptation to the pulmonary environment (79). Thus, through secretion of IL-10, TGF-β, and AREG, lung Tregs modulate local immunity, engage epithelial and mesenchymal cells, and facilitate epithelial repair following injury or infection.

#### Dysregulated Treg responses in lung-specific diseases

In asthma, Treg dysfunction contributes to persistent inflammation and airway remodeling. Tregs in asthma patients often exhibit reduced suppressive capacity, skewed toward Th2 and Th17 responses. Type 2 cytokines (IL-4, IL-5, and IL-13), and IL-6 promote airway inflammation and tissue damage in asthma patients (72, 80). BCL6-expressing Tregs suppress Th2 responses by downregulating GATA3 within Tregs, thereby limiting IL-4 and IL-13 production by Th2 cells (81). Lung Tregs cells can suppress DC function by down-regulation of the expression of glucocorticoid-induced tumor necrosis factor receptor ligand, GITRL, which in turn limits asthma by inducing the apoptosis of pathogenic Th2 and Th17 cells (82). The imbalance of Th17 and Tregs has been shown to contribute to asthma development (83). IL-4-induced STAT6 activation could convert induced Tregs (iTregs) into pathogenic Th17 cells and exacerbate airway inflammation via the GRB2/IL-6 axis, which in turn upregulates Notch4 and downstream Hippo pathway and demethylation of CNS2 in the *Foxp3* locus (84). Targeting the IL-6R–STAT6 axis and Notch4 signaling has demonstrated preclinical efficacy in suppressing airway inflammation and remodeling in asthma models, with early clinical observations supporting IL-6R blockade in asthma patients (85–87). Tregs were demonstrated to improve airway remodeling and restore Th1/Th2 balance through the DLL4-Notch signaling pathway (88). Tregs can be modulated by the androgen receptor signaling, which can downregulate ST2 receptor expression and reduce allergen-induced IL-33 expression in airway epithelial cells (89). Tregs prevent airway inflammation by producing anti-inflammatory cytokines such as IL-10 and TGF-β.

Dysregulated Tregs play a crucial role in the pathogenesis of allergic asthma. In asthma patients, it is reported that CRTH2^+^ circulating Tregs are characterized by increased IL-4 production and reduced immune suppressive function (90), while CCR6^+^ Tregs are more likely to differentiate into pathogenic Th17-like cells, contributing to the pathology of allergic asthma (91). However, restoring functional Treg activity can counteract such imbalance. Indeed, adoptive transfer of ovalbumin (OVA)-specific Tregs into the OVA-sensitized mice attenuated airway hyper-responsiveness and reduced the recruitment of eosinophils, and Th2 cytokine expression in the lung following allergen challenge (92).

Chronic obstructive pulmonary disease (COPD) pathogenesis also involves an imbalance between Tregs and Th17 cells, exacerbating airway inflammation and contributing to the initiation and development of inflammatory responses (93). The deficiency of Tregs leads to the downregulation of IL-10 and TGF-β, which favors the differentiation of Th17 cells and the production of Th17 cytokines such as IL-17A, IL-17F, and IL-22. Th17 cells could activate the lung epithelial cells to produce neutrophil growth factor GM-CSF, chemokines CXCL1 and CXCL18 to recruit neutrophils into the airway.

While the most common treatment of COPD is the use of long-acting β2-agonists and anticholinergic drugs to improve airway obstruction, the combination of anti-inflammatory treatment with inhaled corticosteroids (ICS) is also used depending on the COPD stage. Erythromycin, an antibiotic drug used in COPD, could decrease the IL-17 and IL-23 levels in both the sputum and serum of COPD stage II-IV patients (94), which was further validated in mouse models to suppress Th17 response and promote Treg response (95). Xuanbai Chengqi decoction (XBCQ), an herbal medicine to treat COPD patients in the exacerbation stage, can restore Treg/Th17 balance, increase lung-resident Treg populations, reduce the expression of pro-inflammatory factors such as IL-1β and TNF-α, inhibit inflammatory cell infiltration, and significantly improve lung injury (96).

In idiopathic pulmonary fibrosis (IPF), the role of Tregs remains controversial, with evidence supporting both protective and pathogenic functions. Some studies report the impairment of Treg function in human IPF patients (97) and murine models (98), suggesting the regulatory role of Tregs in IPF. In contrast, other findings highlight that specific Treg subsets may contribute to disease progression. For example, an increased population of activated Tregs has been observed in severe IPF, and Sema7a^+^ Tregs despite being activated, exhibit impaired suppressive capacity and drive TGFβ1-mediated fibrotic responses (99). In line with this, reversing Treg differentiation by PD-1 blockade ameliorates fibrosis in an *in vitro* system (100). On the other hand, functional Tregs have demonstrated protective roles in multiple fibrotic settings. A recent study demonstrated that IL-33 induced the Tff1-expressing Tregs and protected lung fibrosis in a bleomycin (BLM)-injured lung model (101). The therapeutic role of Tregs in lung fibrosis was further validated in the murine pulmonary fibrosis model (102). The authors demonstrated that the adoptive transfer of Tregs after a BLM challenge significantly attenuated pulmonary fibrosis, suppressed the production of fibroblast growth factor 9-positive cells, and reverted the BLM-induced plasma IL-10 expression to basal levels. These studies highlight the therapeutic potential of restoring or enhancing Treg function in IPF and support further investigation of Treg-targeted strategies for fibrotic lung disease.

#### Therapeutic strategies to harness lung Tregs

Given the central role of Tregs in controlling immune homeostasis in the lung, therapeutic strategies that enhance Treg quantity and function hold great promises for treating autoimmune and inflammatory lung diseases. These approaches include cell-based therapies involving the adoptive transfer of Tregs, as well as non-cell-based strategies aimed at boosting endogenous Treg populations. Non-cell-based methods have been explored across multiple pulmonary indications. For example, in asthma, allergen-specific immunotherapy has been shown to enhance the expansion and function of allergen-specific Tregs (103). Other precision approaches, such as targeting the IL-6 receptor (85) and the Notch4 pathway (87), can also restore Treg stability and suppress Th2/Th17-driven inflammation. IL-2 immune complexes (IL-2C), composed of IL-2 and the anti-IL-2 monoclonal antibody JES6-1, have been shown to selectively expand Tregs without broadly activating effector cells, with demonstrated therapeutic efficacy in asthma (104), acute lung injury (105), and COPD (106). In addition, androgen receptor modulators have been shown to modulate ST2^+^ Treg responses by dampening allergen-induced IL-33 production, suggesting a novel hormonal axis influencing Treg function in airway inflammation (107).

Cell–based therapies utilizing Tregs are also under clinical evaluation for severe inflammatory lung conditions. For example, early-phase clinical trials have demonstrated the safety and feasibility of administering cord blood–derived Tregs in patients with COVID-19 associated acute respiratory distress syndrome, with preliminary evidence of survival benefit (108, 109). As cell therapy technologies evolve and off-the-shelf manufacturing becomes more scalable, Treg-based cell therapies may become increasingly accessible for a wider range of lung inflammatory diseases. A major challenge will be designing Treg products that can navigate the heterogeneous and dynamic lung microenvironment, balancing their immunosuppressive and reparative roles while minimizing the risk of promoting fibrosis.

### Tregs in skin and hair

#### Skin-resident Tregs in immune homeostasis and tissue integrity

The skin is a dynamic barrier organ exposed to constant microbial, mechanical, and environmental stress. To preserve tolerance and limit aberrant inflammation, the skin maintains a dense population of tissue-resident Tregs, comprising up to 20% of CD4^+^ T cells in healthy human skin (110, 111). The key skin-homing receptor-ligand axes are CCR6-CCL20, CCR4-CCL22/CCL17, CLA-CD62E, and CCR5/CCR8-CCL4/CCL5 (112–114), enabling precise localization of lymphocytes to cutaneous sites including hair follicles, dermis, and epidermis.

Beyond their classical immunosuppressive roles, skin Tregs perform key non-suppressive functions that support tissue integrity. A well-defined example is their interaction with hair follicle stem cells (HFSCs) via the Jagged-1–Notch signaling axis, which promotes HFSC proliferation and hair regeneration (115). Skin Tregs also help orchestrate immune tolerance to commensal microbes and local self-antigens, integrating antigenic signals with tissue repair and renewal processes (116). Multiple studies have demonstrated that skin-resident Tregs interact with other cell types such as dermal fibroblasts and dendritic cells for their maintenance and proliferation (117–119). Transcriptional profiling reveals skin Tregs are enriched for effector-like molecules (e.g., CD25, ICOS, and CTLA-4) and exhibit a memory phenotype (CD45RO^+^ CD27^+^), with transcriptional regulators like RORα shaping their identity (111, 120).

Upon skin injury, Tregs rapidly accumulate at damaged sites to regulate inflammation and initiate repair (121–123). These cells secrete AREG, activating the EGFR pathway in epithelial and stromal cells to accelerate re-epithelialization (124). Shime et al. demonstrated the role of AREG-expressing Tregs in promoting wound healing upon ultraviolet-B exposure (125). In another study, Nosbaum et al. showed the role of EGFR pathway in mediating Tregs’ function in wound healing (121). These findings suggest that skin Tregs use the AREG-EGFR tissue repair pathway to facilitate wound healing after injury. Tregs also activate latent TGF-β via αvβ8 integrin, inducing neutrophil-attracting chemokines like CXCL5, which confer protection against bacterial invasion at barrier breaches (126). In parallel, Tregs limit excessive leukocyte infiltration and fibrosis by modulating innate immune cells and dermal fibroblasts.

Skin-resident Tregs are polarized toward a Th2 phenotype expressing GATA-3 and restraining pro-fibrotic responses (124, 127). This fine-tunes the balance between tissue regeneration and fibrosis control, particularly in chronic wounds or repetitive damage contexts.

#### Treg dysfunction in autoimmune skin and hair diseases

Deficiencies in the number, stability, or tissue trafficking of Tregs have been increasingly implicated in the pathogenesis of diverse autoimmune skin disorders, where they play critical roles in maintaining immune tolerance, controlling inflammation, and supporting tissue repair.

In psoriasis, Tregs are numerically preserved in the skin but exhibit impaired function. This dysfunction involves multiple mechanisms, including elevated microRNA-210 expression that suppresses Foxp3, an imbalance favoring Th17 cells over Tregs, cytokine-driven conversion of Tregs into Th1- or Th17-like cells (mediated by IL-6, IL-21, and IL-23), and defects in Treg trafficking to the skin (128–134). Recent work identified the polyamine-catabolizing enzyme SSAT1 as a driver of Treg dysfunction in mouse psoriatic skin, where 4-1BBL^+^ keratinocytes induce a Th17-like non-suppressive phenotype in Tregs, while reversible by SSAT1 inhibition (135). Several current and emerging therapies appear to restore Treg function, enhance their frequency, or rebalance the Th17/Treg axis (136). These include the anti-TNF-α antibodies (137), folic acid analogue methotrexate (138, 139), vitamin D treatment (140, 141), retinoids treatment (142, 143), and phototherapy (74, 144, 145). Given the Th17-driven nature of psoriasis, biologic agents targeting IL-23 or IL-17 have shown strong therapeutic efficacy (146–149). In recent studies, the potential of adoptive Treg cell therapy in psoriasis treatment was explored by developing a novel platform for local delivery of cell product and showed substantial amelioration of psoriasis in a mouse model (150), and further therapeutic potential was demonstrated using a CTLA-4 signaling peptide (dNP2-ctCTLA-4) in psoriasis by augmenting Treg/Teff ratio (151).

In hidradenitis suppurativa (HS), a pronounced Th17/Treg imbalance underlies disease pathology (152, 153). Consistent with the role of Th17/Treg cell axis in HS pathogenesis, therapies that have shown clinical benefits in treating HS often act by modulating this axis or by restoring the Th17:Treg ratio (153, 154). Biologics targeting TNF-α, IL-1, IL-12/23, and IL-17 have been used to rebalance the Th17/Treg axis (155–163). However, complete and lasting clinical responses with biological drugs are rarely achieved (155, 163). The CAR-Treg cell therapy has the potential to restore immune cell balance to achieve long-term immune tolerance and ultimately persistent clinical responses.

In systemic lupus erythematosus (SLE), cutaneous involvement is often associated with Treg deficiency (164–166). Multiple experimental approaches are currently being explored for SLE, aiming to restore immune tolerance by leveraging Tregs’ suppressive function which includes rapamycin/retinoic treatment, corticosteroid therapy, tolerogenic dendritic cells, histone peptide tolerance, low-dose IL-2 therapy, antigen-specific nanoparticles, and autoantigen-specific TCR-Treg or CAR-Treg cell therapies (166). Given the central role of Tregs in restraining autoreactive B and T cell responses, reconstituting stable, disease-relevant Treg populations in SLE may help suppress autoantibody production and halt end-organ damage.

In alopecia areata (AA), genome-wide association studies have implicated polymorphisms in Treg-regulating genes (167), suggesting a link between aberrant Treg function and the development of AA. In consistent with this, a significant reduction of the suppressive CD39^+^ Tregs was reported in AA patients (168). The therapeutic value of targeting Tregs in AA treatment, however, has been controversial. A low-dose IL-2 therapy has shown promising efficacy in a prospective open pilot study in 5 patients with severe AA (169), but failed to show efficacy in a larger follow-up clinical trial. The authors reasoned that this might partially be explained by limited expansion of the effector and memory Tregs expressing skin-homing receptors (170). These results suggest that specific Treg populations with tissue-homing capabilities could be critical for re-establishing tolerance in the setting of tissue-specific diseases. Another study explored the therapeutic potential of selective Treg expansion using the IL-2 cytokine antibody complex in C3H/HeJ mice AA model (171). The authors showed that, although intradermal injection of IL-2/anti-IL-2 antibody complex (IL-2C) induced selective proliferation of Jagged-1^+^ Tregs, they failed to induce anagen or reverse the established AA. Therefore, more studies are essential to thoroughly understand the therapeutic potential of Tregs in AA, and further investigations are required to leverage Treg capability for hair regeneration to treat AA.

Vitiligo pathobiology is associated with the auto-reactive T cells that recognize MART-1 (Melanoma antigen recognized by T cells) and other melanosomal proteins as autoantigens (172). Many studies reported that Treg deficiency and impaired skin migration, due to reduced CCL22 expression, are hallmarks of vitiligo pathogenesis (112, 173–180). Therapeutic strategies to augment Treg numbers and functions therefore provide strong promise for vitiligo treatment. Both the adoptive transfer of Tregs and the use of rapamycin could induce a lasting remission of vitiligo in murine disease models (181, 182). Strategies to enhance Treg skin homing, such as cutaneous overexpression of the chemokine CCL22, have shown promise in promoting local immune regulation and could be leveraged for vitiligo therapy (183). Building on this concept, a recent study demonstrated the therapeutic potential of antigen-specific CAR-Treg therapy in vitiligo (184). Researchers engineered CAR-Tregs targeting Ganglioside D3 (GD3), a melanocyte-enriched antigen found in perilesional epidermal cells, and tested them in a TCR transgenic mouse model of spontaneous vitiligo. Compared to polyclonal Tregs, GD3-specific CAR-Tregs conferred significantly greater disease control, highlighting the advantage of antigen specificity in re-establishing immune tolerance in skin autoimmunity.

#### Therapeutic outlook for skin Treg modulation

Given the accessibility of skin and the mechanistic understanding of Treg roles in barrier regulation, autoimmune skin diseases are among the most promising targets for Treg-directed therapies. Emerging cytokine-based strategies, such as low-dose IL-2 therapy have been shown to restore the Th17/Treg immune balance and provide preliminary clinical efficacy for psoriasis in 2 recent clinical trials (185, 186). A more targeted approach, the Treg-selective IL-2 receptor agonist rezpegaldesleukin (REZPEG), has demonstrated promising phase 1b results in patients with psoriasis (NCT04119557) and atopic dermatitis (NCT04081350) (187). In addition, multiple clinical studies using low-dose IL-2 therapy have demonstrated promising results in SLE, which is associated with improved lupus disease activity and reduced autoantibody production (166). However, the clinical efficacy of these approaches may depend not just on expanding Tregs broadly, but on selectively promoting memory Treg subsets with the appropriate tissue-homing capacity needed to exert durable effects at sites of inflammation.

Cell-based therapies are advancing rapidly. In psoriasis and vitiligo, adoptive Treg transfer and engineered local delivery of Tregs or Treg-enhancing agents have demonstrated efficacy in preclinical models. A CTLA-4–mimetic peptide (dNP2-ctCTLA-4) has also improved the Treg/effector balance and alleviated inflammation in psoriasis (151). In HS, a phase 1 trial of a citrullinated protein–targeting CAR-Treg product (SBT-77-7101). In addition, several preclinical studies in SLE patients showed antigen-specific Treg cell engineering as a promising therapy for suppressing SLE, such as using the Smith (Sm) autoantigen specific TCR-Tregs (188) and anti-CD19 CAR-Tregs overexpressing Foxp3 (189). In vitiligo, GD3-specific CAR-Tregs demonstrate superior lesion control versus polyclonal Tregs. In AA, further refinement of strategies that expand Tregs with skin-homing capacity will be critical to achieving durable responses (184). Thus, across autoimmune skin disorders, CAR-Treg cell therapy holds significant promise for restoring immune balance and promoting tissue homeostasis.

Altogether, therapeutic strategies that combine Treg-enhancing signals (e.g., IL-2), tissue-homing optimization (e.g., CLA and CCR6), and engineered antigen specificity are likely to offer the greatest precision and efficacy. Combination therapies integrating Treg modulation with cytokine blockade or microbiome restoration may further optimize disease control while preserving tissue repair functions. Future work should focus on refining Treg delivery platforms, minimizing instability, and balancing their suppressive and regenerative functions for durable immune tolerance in skin and hair autoimmune diseases.

### Tregs in joints

#### Tissue-specific adaptation of Tregs in the joint

In healthy joints, Tregs help maintain tissue homeostasis beyond their classical suppressive roles. They contribute to skeletal integrity by inhibiting osteoclastogenesis, thereby preventing excessive bone resorption and supporting joint architecture (190). Additionally, Tregs interact with stromal and mesenchymal cells in the synovium to regulate low-level inflammation, promote immune quiescence, and maintain tissue-resident immune balance under mechanical stress (191). These joint-protective functions are tightly coordinated and essential for preserving joint health throughout life.

Effective migration and retention of Tregs within inflamed joints are essential for their immunoregulatory function, and understanding the dynamic regulation of these processes is important for informing therapeutic strategies. Tregs show diurnal accumulation in inflamed joints, thereby conferring a time-of-day variation in arthritis severity. Treg numbers peak during the night, coincident with the observed nadir in inflammation. It has been suggested that these increased Treg numbers are not a consequence of elevated cell proliferation within inflamed joints, but more likely a consequence of increased recruitment and/or retention and survival (192). Joint-infiltrating Tregs rely on adhesion molecules (e.g., selectins and integrins) and chemokine receptors like CCR6, which facilitates Treg recruitment in response to high local CCL20 levels, a chemokine also elevated during Th17-mediated inflammation (193, 194).

Recent studies show that Treg migration is metabolically programmed: activation of glucokinase (GCK) and the PI3K–mTORC2 axis drives glycolysis-dependent motility, essential for their infiltration into inflamed tissues (195). GCK-deficient Tregs are functionally suppressive but fail to migrate to sites of inflammation, whereas enhanced GCK activity has no effect on immunosuppressive function but leads to increased motility. More recently, several novel mechanisms that regulate Treg migration have been identified. In a model of collagen-induced arthritis (CIA), IL-6 was shown to induce post-translational modifications of vasodilator-stimulated phosphoprotein (VASP), which resulted in diminished Treg cell trafficking (196).

#### Treg dysfunction and instability in autoimmune arthritis

In RA, juvenile idiopathic arthritis (JIA), and psoriatic arthritis (PsA), Treg numbers in inflamed synovial fluid (SF) are often elevated yet inflammation persists - a phenomenon termed the “Treg paradox” (197). This paradox underscores the presence of numerically abundant Tregs at the site of inflammation, but with insufficient suppressive function. A meta-analysis revealed that while the overall proportion of Tregs defined by CD25 or FOXP3 expression may not differ significantly between RA patients and healthy individuals, circulating FOXP3^+^ CD25^+^ Tregs are reduced in RA and correlate with disease severity (198).

Tregs isolated from RA SF exhibit an activated phenotype (CD69^+^ CD62L^−^), yet display a low proliferative profile, with only a small subset expressing Ki67, a marker of active cell cycling (199). This suggests an accumulation of highly activated but non-proliferating Tregs in the inflamed joint microenvironment. Similar phenotypes and dynamics have been observed in mouse models of inflammatory arthritis, as well as in patients with PsA and JIA, reinforcing the concept that despite numerical presence Treg dysfunction contributes to the failure of immune regulation in the joint (197). This functional impairment of joint-infiltrating Tregs is thought to contribute to the breakdown of self-tolerance and sustained autoimmune inflammation in RA.

An imbalance between Treg and Th17 cells has been demonstrated in multiple inflammatory conditions, including RA and JIA, resulting in increased Th17 frequencies and a pathogenic shift toward Th17 predominance (200). Members of the Ikaros zinc finger family of transcription factors, including Ikaros (encoded by *IKZF1*), Helios (*IKZF2*), Aiolos (*IKZF3*) and Eos (*IKZF4*), have been shown to regulate Treg differentiation, function and stability (201). The expression levels of Helios and Aiolos are significantly reduced in Tregs from RA patients as compared to healthy individuals and those changes correlate with disease activity (202). The reduction of Helios and Aiolos might enhance Treg instability and provide an explanation for their decreased immunosuppressive function (202). Th17 cells arising from trans-differentiation of Tregs losing FOXP3 expression under inflammatory conditions accumulate in inflamed joints and play a key role in the pathogenesis of autoimmune arthritis. The generation of these ex-Tregs in a CIA model is mainly driven by synovial fibroblast-derived IL-6 (203). These Th17-like ex-Tregs express receptor activator of NF-κB ligand (RANKL) known as TNFSF11 leading to the induction of osteoclast differentiation and subsequent destruction of the extracellular matrix and juxta-articular bone resorption (203).

#### Therapeutic potential of Tregs in joint-specific autoimmunity

Despite their dysfunction in arthritis, Tregs retain significant therapeutic potential through both their immunosuppressive and tissue-protective functions. RA patients have diminished Treg migration to inflamed sites, which could be explained by a lack of G-protein-signaling modulator 2 or VASP activity, despite abundance of Tregs in the inflamed joints, suggesting that additional mechanisms are necessary to ensure efficient resolution of inflammation (204). High levels of IL-6 are detected in the peripheral blood and synovial tissue of RA patients, and blocking IL-6R restored VASP phosphorylation and improved Treg migration in RA. Tregs have also been shown to inhibit the differentiation of osteoclasts both in mice (190) and humans (205), and the adoptive transfer of antigen-specific Tregs in a CIA model reverses inflammation and hinders disease progression (206). Taken together, these suggest that enhancing Treg function is a promising strategy to combat inflammatory arthritis. Several treatments, including monoclonal antibodies targeting TNF-α (Infliximab, Adalimumab, and Golimumab), the IL-6R (Tocilizumab) and the IL-1R (Anakinra) indirectly impact on Treg function which might account for their limited efficacy (207). Currently, extensive effort is being dedicated to developing safe and efficient adoptive Treg cell therapies, which need to address Treg instability under inflammatory conditions and ensure the transferred cells do not accelerate disease progression by converting into pathogenic ex-Treg cells. In 2024, a Phase 1 study to evaluate the safety and proof-of-mechanism of an autologous CAR-Treg cell therapy, SBT-77-7101, in patients with refractory RA, was initiated by Sonoma Biotherapeutics.

Thymus-derived nTregs prefer to accumulate in inflamed joints and trans-differentiate to Th17 cells upon stimulation from inflamed synovial fibroblasts whereas iTregs retain FOXP3 expression and regulatory function (208). In addition, iTregs inhibit the proliferation, inflammatory cytokine production, migration, and invasion ability of CIA-synovial fibroblasts *in vitro* and *in vivo*. This suggests that iTreg manipulation may have a greater potential than nTregs for prevention or treatment of RA patients (208).

Although osteoarthritis (OA) is not classically autoimmune, accumulating evidence points to a role for chronic, low-grade inflammation in its progression, particularly within the synovial microenvironment. In this context, Tregs have been implicated in resolving joint inflammation and protecting cartilage integrity. A recent study demonstrated that intradermal injection of lipid nanoparticles (LNPs) co-loaded with type II collagen (Col II) and rapamycin (LNP-Col II-R) into mice with OA effectively induced Col II–specific Tregs, leading to a marked reduction in pro-inflammatory cytokines within the joint (209). This treatment also inhibited chondrocyte apoptosis and cartilage matrix degradation, ultimately alleviating joint pain. These findings highlight the potential of local, antigen-specific Treg induction as a therapeutic approach even in non-autoimmune inflammatory joint disease. They also highlight the need to identify joint-homing receptors and synovial niche signals that support long-term Treg retention, as these insights are key to optimizing targeted Treg therapies in autoimmune joint diseases.

### Tregs in the CNS

#### Treg adaptation and functions in neurorepair and regeneration

The CNS has historically been considered an immune-privileged site due to the blood-brain barrier (BBB) and limited lymphatic drainage. However, the CNS is not completely immune cell free as there are resident myeloid cells such as parenchymal microglia and perivascular macrophages (210), and CD4^+^ T cells help the maturation of microglia (211). Moreover, CNS-derived self-antigens skew lymphocytes toward regulatory phenotypes to establish the immune tolerance in the CNS (212), which corroborates the presence of immunological activities occurring in the CNS at steady state.

While the CNS parenchyma is largely devoid of lymphocytes under homeostatic conditions (213), the meninges harbor resident Tregs that act as immune sentinels, suppressing IFN-γ responses and preventing lymphocyte infiltration into the parenchyma (214). Meningeal Tregs also contribute to short-term memory formation by protecting the hippocampus from inflammation-induced damages (214). In addition, recent evidence reveals that meningeal Tregs can modulate pain sensitivity through a non-immunosuppressive mechanism, specifically by producing enkephalins that act on delta opioid receptors in MrgprD^+^ sensory neurons, establishing a sex hormone-dependent circuit of Treg-mediated nociception control (215). In contrast, parenchymal Tregs typically accumulate during inflammation or injury, where they support remyelination and neurorepair through trophic factor secretion and direct interactions with glial cells (216).

Importantly, Tregs in the CNS are shaped by local cues distinct from peripheral tissues. Meningeal Tregs limit IFN-γ responses and protect hippocampal structure, thereby supporting cognitive function. During injury or infection, self-antigen presentation by CNS-resident cells (e.g., microglia and perivascular macrophages) promotes the accumulation of antigen-specific Tregs, reinforcing local tolerance. However, the relative paucity of professional antigen-presenting cells and the metabolic constraints of the CNS pose unique challenges for Treg activation, retention, and stability (212, 214).

Beyond immune regulation, CNS-adapted Tregs support repair and remyelination. Treg-secreted AREG and keratinocyte growth factor have been implicated in promoting oligodendrocyte precursor cell differentiation and myelin regeneration (216), and Treg AREG also acts directly on astrocytes to suppress reactive gliosis and enhance neurological recovery following ischemic brain injury (217). The pro-remyelinating role of Tregs requires the expression of adhesion molecules such as MCAM and ITGA2 for these contact-dependent regenerative interactions (218). Treg aging impairs this regenerative axis, highlighting the importance of Treg heterogeneity and functional state in CNS tissue repair (218).

#### Treg dysfunction in CNS autoimmunity

In multiple sclerosis (MS), a prototypical autoimmune demyelinating disease, pathogenic lymphocytes infiltrate into the CNS from the blood, and Tregs also accumulate in the cerebrospinal fluid and lesion due to the inflammation in the CNS (219, 220). During neuroinflammation, Tregs infiltrating the CNS parenchyma display impaired suppressive function (221, 222) and reduced Foxp3 expression (223), thereby contributing to disease progression in conditions such as MS. In experimental autoimmune encephalomyelitis (EAE), the mouse model of MS, Tregs in the CNS as well as periphery similarly lose stability and acquire effector-like phenotypes under inflammatory pressure. These Tregs may begin to express IFN-γ and lose regulatory identity, contributing to inflammation (224).

Oxidative stress plays a major role in this dysfunction. Elevated mtROS impair Treg survival in the CNS during autoimmunity (225). Scavenging mtROS improves Treg persistence and mitigates EAE severity. Given that Tregs are impaired in the context of autoimmunity, it is tempting to restore Treg cell function by adoptively transferring *ex vivo*-expanded Treg cells. Indeed, an adoptive transfer of Tregs can attenuate EAE progression (226), and IL-10 secreted by Tregs is a critical regulator of the CNS inflammation (227, 228).

Despite promising results from adoptive transfer studies, a major translational barrier remains: the delivery of Treg-targeted therapies across the BBB. Systemically administered Treg therapies, such as IL-2 muteins or expanded Tregs, exhibit poor CNS penetration under homeostatic conditions (219, 226, 229). While inflammation can transiently increase BBB permeability, it also poses safety concerns by permitting off-target effects in demyelinated or degenerating neural tissues. Overcoming these challenges is essential to unlock the full therapeutic potential of CNS-resident Tregs in diseases like MS.

#### Emerging therapeutic strategies for CNS-specific Treg targeting

Restoring or enhancing Treg function holds substantial therapeutic promise in MS and related conditions. Several translational approaches are under development. Engineered antigen-specific Tregs targeting myelin-derived antigens such as myelin oligodendrocyte glycoprotein (MOG) or myelin basic protein (MBP) have shown strong clinical promise. These include both CAR-Tregs and TCR-Tregs, some of which have reached preclinical validation (230–233). There continues to be advances in Treg-based platforms aimed at CNS autoimmunity, with a focus on MS (66, 229).

As a non-cell-based approach, tolerogenic vaccines utilizing mRNA-based antigen expression (234) or glycosylated antigen (235) have shown that immune tolerance can be established *in vivo* to treat EAE. Alternatively, bioengineered tolerogenic microparticles delivering IL-2 muteins, rapamycin, and MHCII loaded with a myelin peptide promote the expansion of Tregs *in vivo* and consequent attenuation of EAE (236). While these strategies are yet to come to clinical trials, they carry the risk of activating pathogenic effector T cells. Overall, these strategies must navigate the unique challenge of achieving CNS access and long-term Treg stability within the inflamed nervous system.

### Tregs in adipose tissue

#### Tissue-specific Treg functions in metabolic homeostasis

Visceral adipose tissue (VAT) harbors a distinct population of tissue-resident Tregs that extend their function well beyond immune suppression. These VAT Tregs actively participate in maintaining systemic metabolic homeostasis. Tregs accumulating in VAT enhance insulin sensitivity by regulating inflammation in the fat tissues, but in obese mice, the number of VAT Tregs is significantly reduced increasing insulin resistance (7, 237–241). Unlike lymphoid Tregs, VAT Tregs express high levels of the IL-33 receptor ST2 and the nuclear receptor PPARγ. The exogenous injection of IL-33 into obese mice can increase insulin sensitivity by promoting VAT Tregs (7, 239, 242, 243). The reduction of VAT Tregs in obese mice is likely attributable to impaired cholesterol metabolism of VAT Tregs within the fat microenvironment (244).

VAT Tregs arise primarily from thymus-derived precursors and undergo tissue-specific adaptation *in situ*, acquiring unique transcriptional, epigenetic, and metabolic signatures. IL-33 acts as a potent trophic factor, enhancing VAT Treg expansion and AREG expression. These cells contribute to local immune regulation by modulating innate lymphoid cells and adipose tissue macrophages, thereby reducing proinflammatory cytokine production and maintaining insulin responsiveness.

#### Functional heterogeneity and context-dependent adaptation

Unlike in obese mice, VAT Tregs in aged mice are not able to regulate age-related insulin resistance despite their increased accumulation (245). The controversial role of VAT Tregs in the context of obesity and aging is likely due to the heterogeneity of the VAT Treg compartment (50, 246). Single-cell and fate-mapping studies have revealed the existence of multiple VAT Treg subsets with distinct transcriptional and metabolic profiles. For instance, CD73^hi^ ST2^lo^ PPARγ^lo^ Tregs dominate in lean mice and promote insulin sensitivity, while CD73^lo^ ST2^hi^ PPARγ^hi^ Tregs accumulate during aging and in obesity but have diminished metabolic benefit (246). This age-associated phenotypic shift suggests that total VAT Treg numbers alone are not predictive of function; rather, subset composition and local cues dictate their impact.

Sex-specific differences further contribute to VAT Treg heterogeneity. In female mice, CXCR3^+^ VAT Tregs represent a dominant population regulated by T-bet and restrained by PPARγ, IL-33, and androgens (247). These cells exhibit enhanced ectonucleotidase activity (CD73 and CD39) and differ functionally from ST2^+^ counterparts that express high levels of AREG, IL-10, and CD25. Such diversity implies that therapeutic strategies must be tailored to the specific VAT Treg subtype relevant to the disease context, whether obesity, aging, or sex-dependent metabolic disorders.

#### Translational insights and limitations

Most mechanistic insights into VAT Tregs come from murine models. Human adipose tissue Tregs also express high levels of PPARγ and immunomodulatory genes like *PRDM1* (encoding BLIMP1) and *CCR4*, but show variability in ST2 expression across studies (7, 248). Moreover, associations between VAT Treg frequency and metabolic disease in humans remain conflicting (248, 249); some report negative correlation with obesity, while others note increased Tregs in obese adipose tissue with impaired function. These discrepancies likely reflect heterogeneity in fat depots, disease stage, or tissue processing methods.

While no clinical therapies currently target VAT Tregs directly, the findings in this tissue suggest that modulating IL-33/ST2 signaling, restoring PPARγ-driven programs, or selectively expanding metabolically beneficial Treg subsets may offer therapeutic benefits in metabolic syndrome, type 2 diabetes, and inflammaging. However, translating murine findings to humans remains challenging due to depot-specific heterogeneity, variable ST2 expression, and inconsistent associations with insulin resistance. Identifying conserved human VAT Treg signatures will be critical for developing targeted interventions without triggering systemic immunosuppression.

## Conclusion

This review highlights the evolving understanding of Tregs as tissue-adapted immune regulators with diverse functions that extend far beyond canonical suppression. In distinct anatomical niches, such as the gut, lung, skin, joints, CNS, and adipose tissue, Tregs integrate local cues to assume specialized roles that include epithelial regeneration, metabolic regulation, barrier maintenance, and control of fibrotic responses. These functions are orchestrated by tissue-specific transcriptional, epigenetic, and cytokine-driven programs, reflecting remarkable cellular plasticity and environmental adaptation. **Figure 1** provides a concise visual summary of the specialized markers and inflammatory contexts shaping Treg functions in each tissue.

However, this specialization renders Tregs vulnerable to dysfunction under chronic inflammation, metabolic stress, or aging, contributing to the breakdown of tissue homeostasis in autoimmune and inflammatory diseases. The instability of tissue Tregs, their conversion into proinflammatory ex-Tregs, and the loss of key homeostatic signals (e.g., IL-2, IL-33, and AREG) are common threads across autoimmune pathologies. As a result, restoring or reinforcing tissue-specific Treg programs has become a central strategy in next-generation immunotherapies.

Recent advances, ranging from polyclonal and antigen-specific TCR- or CAR-Treg therapies to IL-2 muteins, tissue-targeted biologics, and tolerogenic vaccines, are now enabling precision modulation of Treg activity in specific tissues. Yet, most of these approaches remain rooted in murine studies, and the translation to human disease contexts remains limited by incomplete knowledge of human tissue Treg phenotypes, homing signatures, and stability requirements.

To advance Treg-based therapies from experimental promise to clinical success, future research should focus on defining stable, functional human tissue Treg subsets, developing engineering strategies to enhance tissue homing, persistence, and non-suppressive functions, incorporating multi-omics and spatial profiling to map Treg interactions with local immune and stromal cells, and designing combinatorial approaches that synergize Treg support with cytokine blockade or metabolic modulation. By embracing the complexity and versatility of tissue-adapted Tregs, we can unlock their full therapeutic potential, not only as suppressors of inflammation but as active architects of immune tolerance, tissue repair, and homeostasis in autoimmune diseases.
